# MiRFinder: an improved approach and software implementation for genome-wide fast microRNA precursor scans

**DOI:** 10.1186/1471-2105-8-341

**Published:** 2007-09-17

**Authors:** Ting-Hua Huang, Bin Fan, Max F Rothschild, Zhi-Liang Hu, Kui Li, Shu-Hong Zhao

**Affiliations:** 1 Key Lab of Agricultural Animal Genetics, Breeding, and Reproduction of Ministry of Education & Key Laboratory of Swine Genetics and Breeding of Ministry of Agriculture, Huazhong Agricultural University, Wuhan, 430070, P R China; 2 Department of Animal Science and Center for Integrated Animal Genomics, Iowa State University, Ames, IA, 50011, USA; 3 Department of Gene and Cell Engineering, Institute of Animal Science, Chinese Academy of Agricultural Sciences, Beijing 100094, P R China

## Abstract

**Background:**

MicroRNAs (miRNAs) are recognized as one of the most important families of non-coding RNAs that serve as important sequence-specific post-transcriptional regulators of gene expression. Identification of miRNAs is an important requirement for understanding the mechanisms of post-transcriptional regulation. Hundreds of miRNAs have been identified by direct cloning and computational approaches in several species. However, there are still many miRNAs that remain to be identified due to lack of either sequence features or robust algorithms to efficiently identify them.

**Results:**

We have evaluated features valuable for pre-miRNA prediction, such as the local secondary structure differences of the stem region of miRNA and non-miRNA hairpins. We have also established correlations between different types of mutations and the secondary structures of pre-miRNAs. Utilizing these features and combining some improvements of the current pre-miRNA prediction methods, we implemented a computational learning method SVM (support vector machine) to build a high throughput and good performance computational pre-miRNA prediction tool called MiRFinder. The tool was designed for genome-wise, pair-wise sequences from two related species. The method built into the tool consisted of two major steps: 1) genome wide search for hairpin candidates and 2) exclusion of the non-robust structures based on analysis of 18 parameters by the SVM method. Results from applying the tool for chicken/human and D. melanogaster/D. pseudoobscura pair-wise genome alignments showed that the tool can be used for genome wide pre-miRNA predictions.

**Conclusion:**

The MiRFinder can be a good alternative to current miRNA discovery software. This tool is available at .

## Background

### An overview of miRNA

MicroRNA (miRNA) is a special class of endogenic RNA molecules that can down-regulate the expression of protein coding genes at the post-transcriptional level by means of incomplete complementary interactions. The biogenesis of miRNA involves several steps: 1) The majority of long primary transcripts of the miRNA genes are transcribed by RNA polymerase II [1,2]; 2) The 7-methylguanosine capped and poly(A) tailed transcripts are cleaved by the nuclear RNase III Drosha to release the precursors of miRNA (pre-miRNA) in the nucleus [3]; 3) The precursors of miRNA that possess a thermodynamic stabile hairpin structure are exported into the cytoplasm by Exportin-5 or HASTY [4-7] and 4) An additional cleavage in the cytoplasm yields 18–23 nt mature miRNA [8-10]. The first two miRNAs, lin-4 and let-7, were discovered as important post-transcriptional regulators for the development of Caenorhabditis elegans in the early larval stage [11]. Since then, considerable effort has been devoted to finding miRNA genes, and to date, numerous miRNAs have been identified. Recent experiments, aimed at elucidation of the function of miRNAs, have confirmed that many miRNAs are involved in potentially many developmental and physiological processes [summarized in additional file 1 table 1].

### Existing approaches for miRNA identification

Systematic miRNA identification was first made by the cloning and sequencing of cDNAs prepared from the approximately 22-nuleotide (NT) fraction of total RNA [12-14]. A number of miRNAs from various species have been cloned by this method. However, the expression levels of miRNAs are quite different in different tissues and at different developmental stages [12]. The expression levels of some miRNAs are too low for easy detection. Moreover, in many cases not all of the tissues and developmental stages were sampled. The majority of miRNAs cloned by this method are abundantly/ubiquitously expressed ones that dominate the extracted RNA products due to technical difficulties.

Computational methods, using newly acquired genome sequences from a variety of species, represent another useful way to avoid these problems in miRNA identification [summarized in additional file 1 table 2]. The conserved structure, phylogenetic shadowing and other features of miRNAs suggest that a computational approach may complement well the direct cloning method. A homology search, which can detect homologues of known miRNAs, was first successfully implemented in miRAlign [15]. With a primary focus on pair-wise genome sequences, combined with some sequence features to distinguish miRNA and non-miRNA hairpins, a number of tools have successfully predicted miRNA genes that display close homology in two species [16-18].

Furthermore, some machine-learning methods, including the SVM method, have been introduced into miRNA prediction and have been used with some success [19-24]. The SVM method was first introduced by Pfeffer et al. [22]. The features they used are simple and straightforward: the free energy of folding, the length of the longest symmetrical stem, the count of A, C, G and U nucleotides in the symmetrical stem, and the number of A-U, G-C and G-U pairs in the predicted minimal energy structure. After training they obtained a model that assigned a positive score to 71% of the true positives and to only 3% of false positives. Another set of novel secondary structure description syntaxes were developed by Xue et al. [21] who used triplet elements to represent the local contiguous structure-sequence information and proposed a set of new parameters. After training with positive and negative datasets, they achieved a level of about 90% accuracy with human data.

In three recent studies, RNAmicro, miRNA SVM and miPred extended the usage of SVM in miRNA prediction [23-25]. Utilizing multiple sequence alignments, Hertel et al. developed a SVM based program, RNAmicro, to predict miRNAs in various organisms [23]. Descriptors introduced into the program include the properties of the hairpin, Z-score related properties and entropy related properties. The tool can be used to recognize microRNA precursors in multiple sequence alignments and has been successfully applied to recent genome-wide surveys of mammals, urochordates and nematodes. The miRNA SVM program introduced by Helvik et al. was based on prediction of 5' Drosha processing sites in hairpins, which are essential for pre-miRNA discovery [24]. The classifier can correctly predict the processing site for 50% of the known human 5' miRNAs. The miRNA SVM program used 18 features including the composition properties of the hairpin and a set of processing site related properties. A definitive effort to compile 29 global intrinsic hairpin folding attributes from the pre-miRNA sequences without relying on the comparative genomic information was performed by Kwang et al. [25]. They characterized a pre-miRNA at the dinucleotide sequence, hairpin folding, non-linear statistical thermodynamics and topological levels. The SVM classifier model was trained on 200 human pre-miRNAs and 400 non-miRNA hairpins, and achieved 93.50% accuracy.

### Motivation of our study

It is commonly recognized that the small miRNA family is quite large. To date, 474 human and 78 fly miRNAs have been discovered, and more are likely to be identified [26]. A major concern in miRNA identification now is the need to improve existing prediction methods and develop new methods for better performance and efficiency.

In a large genome, there are many sequence segments that can fold into hairpin secondary structures similar to pre-miRNA. However, pre-miRNAs are only a very small proportion of these sequence segments. Therefore, distinguishing between miRNA and non-miRNA hairpins is crucial in the computational identification of miRNAs. The hairpin structure of pre-miRNA is a good feature for miRNA prediction, but hairpin structures are not unique to miRNAs. The short length of pre-miRNA sequences, with low specificity relative to the overwhelming number of genome background sequences, makes genome-wide miRNA prediction complicated. The majority of the non-miRNA hairpins residing in a genome can be removed by genome comparisons. The drawback of this method is that multiple genome alignment is computationally intensive. In addition, the existing packages using multiple alignments that detect pre-miRNA candidates may lose real pre-miRNAs that are less conserved or conserved only between two species. On the other hand, the pair-wise genome alignments are relatively easy to implement.

Combining previously published work, our analyses of the pre-miRNA sequences indicated that the current knowledge of the secondary structure and the mutation characteristics of the pre-miRNAs are incomplete. Comparative analyses and computer simulation revealed a set of mutation-related features valuable for pre-miRNA prediction. Based on the evaluation of the features discovered so far, we have improved the syntax to describe the stem-loop structure for effective miRNA prediction and developed a new tool, miRFinder, which uses a comprehensive combination of many well-selected parameter measurements for improved miRNA prediction. Here we report our successful *in silicon *prediction of pre-miRNA candidates using miRFinder.

## Implementation

### Vectors representing the features of pre-miRNA

**Figure 1:**
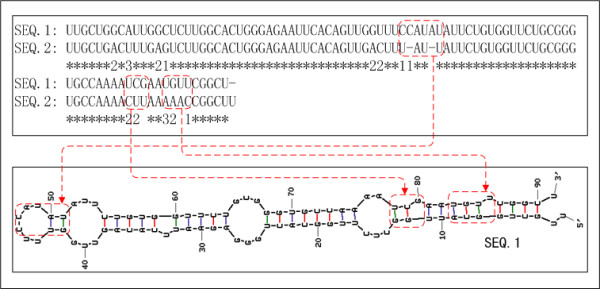
Mutation profile of miRNA. There are three types of mutations that cause slight disturbance of the secondary structure of pre-miRNA: (1) mutations in the loop region; (2) mutations between A and G, U and C in the stem region; (3) mutations in the interrelated region of both arms that do not break the base-pairing. The three types of mutations are marked by the numbers 1, 2 and 3, respectively, under the alignments. The conserved nucleotides are marked as "*".

The miRFinder tool improves the ability to distinguish between miRNA and non-miRNA hairpins by improving the representation of the sequence and structure features of pre-miRNA. Our investigation showed that the relationship between mutation patterns and the secondary structures of pre-miRNA are significantly distinct from that of non-miRNA hairpins. According to most literature, the pre-miRNA coding arm suffers the highest selective pressure, followed by the non-coding arm, stem region, loop region, and flanking sequence. A mutation on the stem region containing the mature miRNA seldom happens [16,27]. Our analysis revealed that 23 out of the 72 conserved pre-miRNAs between D. melanogaster and D. pseudoobscura have mutations in the stem region. We also found a large number of similar pre-miRNAs that have mutations in the stem region in the human, the mouse and other organisms. Further analysis showed that all of these mutations have only slight changes in the secondary structure of pre-miRNA (Figure 1). We call them neutral mutations. Theoretically, mutations between A and G, and U and C suffer relatively lower selective pressure due to the compatibility of G-U base-pairing in pre-miRNA during evolution, which may increase their mutation frequency (Table 1, pMutFeq). Unfortunately the mutation frequencies between A and G, and U and C are not sufficiently different to distinguish the miRNA and non-miRNA hairpins. This is due mainly to the relatively short length of pre-miRNA and the masking effect of the inherent high mutation frequency between A and G, and U and C. However, in non-miRNA hairpins the disturbance of the secondary structure and MFE resulting from mutations is much higher than that of real pre-miRNAs (Table 1, pVStrc and pVMFE). The mutation types of pre-miRNAs and their influence on the secondary structure are valuable features for pre-miRNA prediction but have been seldom used for prediction.

**Table 1:** Test results of the 18 parameters implemented in miRFinder

**Parameters**		**Pre-miRNA**		**Non-miRNA Hairpins**		**Parameter Test**	
ID	Symbol	Mean	Std. Deviation	Mean	Std. Deviation	F value	T test

01	pMFE	-0.444	0.072	-0.218	0.086	1.44	0.000
02	pVMFE	0.455	0.936	0.506	0.632	0.03	0.014
03	pVStrc	0.903	1.812	3.635	4.703	0.41	0.000
04	pMatch	0.892	0.063	0.639	0.176	1.06	0.000
05	pDI	0.050	0.058	0.088	0.126	0.21	0.000
06	pMismatch	0.097	0.060	0.196	0.119	0.55	0.000
07	pBulge	0.014	0.036	0.169	0.209	0.64	0.000
08	pMutFreq	0.018	0.024	0.136	0.126	0.78	0.000
09	"=-"	0.043	0.029	0.035	0.031	0.13	0.000
10	"=="	0.649	0.081	0.466	0.089	1.08	0.000
11	"=:"	0.090	0.041	0.093	0.037	0.04	0.001
12	"--"	0.023	0.041	0.043	0.076	0.17	0.000
13	"-="	0.043	0.029	0.035	0.031	0.13	0.000
14	"^^"	0.008	0.021	0.090	0.130	0.54	0.000
15	"^="	0.014	0.018	0.044	0.026	0.69	0.000
16	"::"	0.044	0.044	0.102	0.086	0.45	0.000
17	":^"	0.014	0.018	0.044	0.026	0.69	0.000
18	":="	0.076	0.041	0.048	0.037	0.36	0.000

Note: T tests are 2-tailed. The F value represents the discriminative power of the parameters. The 18 parameters were coded as. 1: Minimum Free Energy; 2: The difference of the MFE of the sequence pair; 3: The difference of the structure of the sequence pair; 4–7: Base pairing and other properties of the 22 mer hypothesized mature miRNA; 8: The mutation frequency of the sequence segment pair; 9–18: The frequency of the 10 possible secondary structure elements (combinations of 2 adjacent characters) in the pseudo code of stem region (represented by the new syntax). [See details in additional file 1].

**Figure 2:**
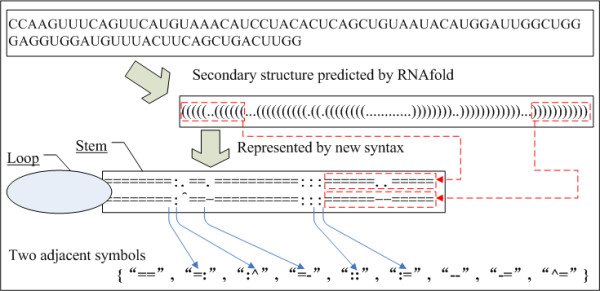
An example of how a hairpin is represented using the new syntax. Symbols of "=", ":", ".", "-"and "^" indicate states of paired, unpaired, insertion, deletion and bulge, respectively. The frequency of each element (combinations of every two adjacent symbols) of the pseudo code of the structure will be used as input vectors for SVM.

Recent reports have shown that local sequence features, such as the distribution of the loops, are distinctly different between that of miRNA and non-miRNA hairpins. We improved the syntaxes proposed by Xue et al. [21] to further elucidate the information of the local secondary structure [see additional file 1 for details of the syntax]. We introduced five symbols "=", ":", ".", "-" and "^" (indicating states of paired, unpaired, insertion, deletion and bulge, respectively) to mark the states of each nucleotide in secondary structure prediction. The new syntax focused on the information of every two adjacent symbols. The frequency of each combination defines a set of novel and useful features (Table 1, Parameters 9–18). As an example, Figure 2 illustrates how a hairpin is represented using the new syntax.

A selection criterion, which has been used by Dror et al. is used to show the discriminative power of these parameters [28] (Table 1). The results show that these parameters represent important features for pre-miRNA prediction.

### Dataset preparation for SVM model training and testing

Construction of the training datasets involved several steps. 1) Construction of positive training subsets. The positive training subsets contained about 4,000 pre-miRNA pairs. The pre-miRNA sequences of human, mouse, pig, cattle, dog and sheep collected from the miRBase (release 8.2) [29] were compared with each other to find the conserved pairs between any two species. The pairs of secondary structure containing multiple loops were eliminated from the datasets. 2) Construction of negative training subsets. The negative training subsets were constructed by the sequence segments extracted from UCSC genome pair-wise alignments (human, mouse) [30]. We used a program that implemented the SW-like algorithm [see the algorithm in additional file 1] to scan the sequence segments that can fold to form hairpin secondary structures. About 10% of the sequence segments were extracted by a stratified selection to generate a subset. The sequences that contained experimentally confirmed pre-miRNAs were eliminated manually. The negative training subsets were constructed by randomly selecting about 4,000 sequence segments from the subset. [See the datasets in additional file 2].

We also created test datasets containing a negative subset simulating the background of the genome sequence and a positive subset containing homolog pre-miRNA pairs. The construction of the negative subset was based on earlier methods for computational problems described in the literature, co-mingling a set of non-miRNA genomic sequences from different species with a set of shuffling sequences [31]. We tried to avoid an unbalanced case study by using a combination of each sequence type (6,193 chicken non-miRNA genomic sequences and 5,000 shuffling sequences). The positive subset (containing 500 homolog pre-miRNA pairs) was generated by a comparison of pre-miRNAs between different species. [See the datasets in additional file 2].

### Development of new tool for pre-miRNA prediction

**Figure 3:**
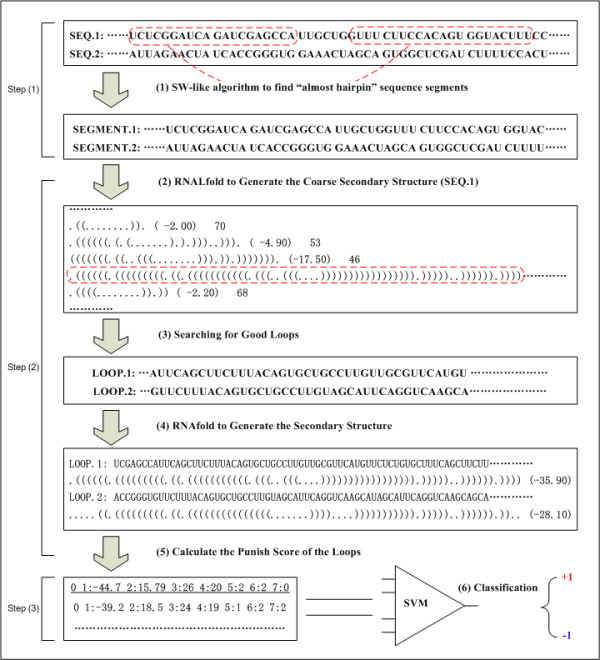
The pipeline of the miRFinder. It consists of 5 steps: (1) Smith-Waterman like algorithm searches the genome of short hairpins; (2) The sequence is folded by RNALfold (Hofacker et al., 2004) to get the local structure; (3) the extended good loops is picked out by schLoop; (4) the good loops are re-folded by RNAfold (Zuker & Stiegler, 1981) to get the MFE and secondary structure; (5) the Punish program calculates the punish score of each paired sequence segments; (6) the sequence is then predicted to be miRNAs or non-miRNA hairpins using the SVM (support vector machine) classifier.

Utilizing the 18 parameters (Table 1), we developed a tool, called MiRFinder, to predict pre-miRNAs that are conserved in two genomes. There are three major steps built into the program (Figure 3). An algorithm based on the Smith-Waterman algorithm [32] was developed to quickly scan the genome pair-wise sequence to get the regions that have high potential to form a hairpin [see additional file 1 for details]. The criteria for the selection were: a) a minimum length of the hairpin of 18 nucleotides (lowest number of base pairings of mature miRNA) and b) no multiple loops. The good loops were folded by a modified version of the Vienna RNA package [33] to get all of the possible secondary structures. Hairpin loops were picked up, and the relevant punish scores corresponding to the 18 parameters were calculated based on the sequence information, MFE and secondary structure. The final classification of pre-mirRNAs from non-mirRNA hairpins was based on excluding non-robust structures by SVM scoring.

The punish scores of 18 proposed parameters of the training datasets (see "dataset preparation for SVM model training and testing" section) were calculated to generate score datasets. The score datasets were split into two subsets (TS1, TS2), one for training and one for cross validation. Each subset included 1,500 positive samples and 1,500 negative samples selected from the score dataset by a random procedure. For each dataset, all parameters were scaled linearly from -1 to 1. The TS1 was used for the SVM model training. A SVM classification program, LIBSVM [34], was trained to generate a model to classify the loops as pre-miRNA or other sequences. A cross validation (CV) technique was used for the selection of the most suitable parameters for training.

## Results and discussion

### MirFinder can accurately distinguish miRNA and non-miRNA hairpins

**Figure 4:**
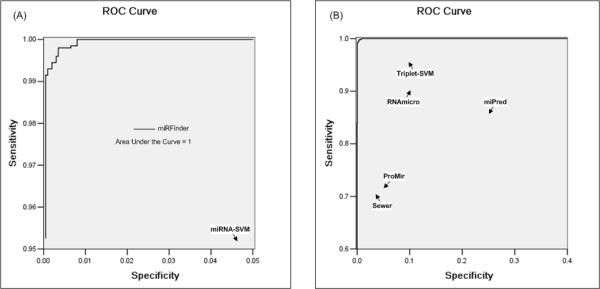
The ROC-curve. The solid line shows the ROC-curve for the miRFinder that was trained on miRNAs versus non-miRNA hairpins. The points for Sewer, ProMir, Triplet-SVM, miRNA-SVM, miPred, and RNAmicro are the sensitivities and specificities reported by Sewer et al. (2005), Nam et al. (2005), Xue et al. (2005), Hertel et al. (2006), Helvik et al. (2007) and Kwang Loong et al. (2007). The sensitivity and specificity of miRScan are 0.5 and 0.7, respectively, and are not included in the figure. Panel (A) is a detailed excerpt of Panel (B).

The training of the model yielded an accuracy rate of 99.6% (radial basis function-kernel, g-0.125 c-32, five folds cross validation). The TS2 subset was subsequently used to test the model. The results show that the model could correctly assign 99.4% of the samples in TS2. The ROC cure analysis of the model showed that the AUC is approximately equal to 1 (Figure 4). The results show that our method had good performance distinguishing between miRNA and non-miRNA hairpins.

### An actual example: testing of the tool with aligned genome data from chicken/human and D. melanogaster/D. pseudoobscura comparisons

To test the performance of the tool in actual prediction, miRFinder was used to predict pre-miRNAs from chicken/human pair-wise genome alignments. The alignments were downloaded from the UCSC bioinformatics site [30]. The program was run on a desktop computer (1.8 GHZ CPU, WindowsXP and 256 M RAM). A total of 222 good candidates were obtained [score>0.9, see additional file 1 figure 3A]. These candidates were aligned to the pre-miRNAs collected from miRBase [29]. A total of 60 matched experimentally confirmed chicken pre-miRNAs were identified [with 86 experimentally confirmed pre-miRNAs that are highly conserved between the chicken and human genomes; the prediction match rate is 70% (60/86), see additional file 1 figure 1A and additional file 3 table 1]. In total, 159 sequence segments with high potential to be pre-miRNAs were detected by miRFinder [see additional file 1 figure 1B and additional file 3 table 1]. The prediction results of the chicken/human genome alignments showed that the tool has good performance. In our experience the tool is easy to operate and does not demand much computing power, thus it may be used for high throughput prediction.

To test whether the miRFinder was suitable for organisms other than vertebrates, it was used to predict pre-miRNAs in D. melanogaster/D. pseudoobscura genome alignments. We obtained 188 good candidates [score>0.9, see additional file 1 figure 3B], of which 34 matched experimentally confirmed miRNAs [see additional file 4 table 2]. With about 73 pre-miRNAs highly conserved between the D. melanogaster and D. pseudoobscura genomes, the prediction results showed that the detection rate was 47% (34/73). Our results suggest that the tool can be implemented in the fly genome, but the performance was apparently worse than in the chicken genome.

### Assessing the tool

In this study, we assessed the miRFinder along with other similar miRNA prediction tools, miRscan and triplet-SVM [21,35]. The miRscan is one of the most well-known and widely used miRNA prediction software designed for miRNA prediction in the C. elegans/C. briggsae genomes [35]. The triplet-SVM classifier is well regarded for distinguishing between miRNA and non-miRNA hairpins in animals, plants and other genomes, and was optimized for the human genome [21]. These tools have relatively good performance. Some other tools also reported good performance, but they are methodologically different or not supported to scan genomes, such as ProMiR, and thus not included in this assessment.

In assessing the tool, two major aspects were taken into consideration: 1) the false discriminative rates (the false positive rate) and 2) the detectable rate (the sensitivity). Each program was run with the test datasets on the default configuration settings.

We used relatively small test datasets (see "dataset preparation for SVM model training and testing" section) to examine the performance of miRFinder and miRscan. The results of the miRFinder and miRscan trials are similar, to some extent. For the negative datasets the false discriminative rates of miRFinder and miRscan were 0.70% (79/11,193) and 0.23% (26/11,193), respectively. Interestingly, 11 sequences were recognized as good candidates by both of the software programs. However, for the positive datasets only 158 (158/500) sequences were recognized as good pre-miRNA candidates by miRScan, while over 99% of these pre-miRNAs were detected by miRFinder. These results are similar to the reports that the application of MiRscan for the C. elegans/C. briggsae genome analysis can detect only half of the 58 previously known miRNAs [35].

For the 11,193 hairpin-like sequences derived from the partial sequences of the chicken genome, over 1,000 were recognized as good candidates by triplet-SVM. This result is similar to the evaluations of triplet-SVM classifier reported by Helvik et al. [24]. Compared with triplet-SVM, miRFinder reduced the number of the candidates to about 10%. Nevertheless, miRFinder was focused on the conserved pre-miRNAs and thus possibly missed the non-conserved pre-miRNAs.

Noticeably, processing a large vertebrate genome for pre-miRNA prediction is time consuming. Test results revealed that miRFinder is faster than miRscan (hundreds of mega-bases per CPU hour compared to several mega-bases per CPU hour, respectively). For example, to process 530 sequences, miRFinder took only 40 seconds while miRscan took 215 seconds [see additional file 1 figure 1E].

## Conclusion

MirFinder can accurately distinguish between miRNA and non-miRNA hairpins. Compared to similar methods, our method has better performance. At sensitivity levels, mirFinder is comparable to methods, such as RNAmicro, that rely on sequence or structure conservation [23]. Furthermore, our method reduces the number of candidates, which makes it more practical than others. A down side might be that the species specific pre-miRNAs could be lost since these miRNAs would be left out in the sequence alignment step before starting the prediction.

## Availability and requirements

Project Name: MiRFinder

Project Home Page: . [Also see the application and source code in additional file 5].

## Competing interests 

The author(s) declares that there are no competing interests.

Operating Systems: All platforms with GNU C++ compiler.

Programming Languages: C++

License: Academic Free License .

Non-academics Restrictions: License needed

## Authors' contributions

SHZ, MFR, BF and KL initiated the project and guided the forming of the ideas. THH developed the method and wrote the source code and implemented most of the experiments under the guide of SHZ. MFR and ZLH provided helpful insight in the method development and helped in the writing and assessment of the manuscript. All authors have read and approved the final manuscript.

## Supplementary Material

Additional file 1 Supplemental document. The document provided supplemental information of the manuscript. Click here for file

Additional file 2 Datasets. This archive contains the training and testing datasets. Click here for file

Additional file 3 Supplemental table 1. Chicken/Human candidate miRNAs predicted by miRFinder. Click here for file

Additional file 4 Supplemental table 2. D.pseudoobscura/D.melanogaster pre-miRNA candidates detected by miRFinder. Click here for file

Additional file 5 miRFinder. This archive contains application and source code of miRFinder. Click here for file
