# Supplementary material for: MiRFinder: an improved approach and software implementation for genome-wide fast microRNA precursor scans
Source: BMC Bioinformatics. 2007 Sep 17;8:341. doi: 10.1186/1471-2105-8-341 (PMC2206061; doi:10.1186/1471-2105-8-341)
Supplement: Additional file 1 — Supplemental document. The document provided supplemental information of the manuscript. [file 1471-2105-8-341-S1.pdf]

Supplementary Materials

Syntaxes describing the information of the local secondary structure

The predicted secondary structure was marked with three symbols "(", ")", and ".", representing the status of each nucleotide base. The "." indicates that the nucleotide base is unpaired. The "(" and ")" indicates two paired nucleotide bases which are located near the 5'-end and near the 3'-end respectively. Taking into account the interaction of the two arms of the stem, five symbols "=", ":", ".", "-" and "^" (indicating states of paired, unpaired, insertion, deletion and bulge, respectively) were introduced into the syntax to represent the status of each nucleotide base pair in the stem. Each of them corresponded to the status of the match, mismatch, deletion, insertion in the interior loop, and insertion in the bulged loop, respectively. The new syntax focused on the information of every two adjacent symbols. There are 14 possible combinations and each combination represents a special meaning. For example: "=-", "=.", and "=:" represent the boundary of the stem/loop; ":^" represents that the loop is asymmetric, etc. The frequency of each combination defines a set of novel and useful features.

Parameters describing features of pre-miRNA

Two sequences of the pair-wise genome segments are represented as:
$SEQa = SEQa_1, SEQa_2, SEQa_3, ..., SEQa_n$ and $SEQb = SEQb_1, SEQb_2, SEQb_3, ..., SEQb_n$.
Secondary structures of the two sequences are represented as:
$STRCa = STRCa_1, STRCa_2, STRCa_3, ..., STRCa_n$ and $STRCb = STRCb_1, STRCb_2, STRCb_3, ..., STRCb_n$.
Two arms of the stems of the master sequences are represented as:
$STEMl = STEMl_1, STEMl_2, STEMl_3, ..., STEMl_i$ and $STEMr = STEMr_1, STEMr_2, STEMr_3, ..., STEMr_i$.
The miRFinder uses 18 parameters describing the features of the pre-miRNA:

(1) pMFE: Minimum Free Energy of the secondary structure. MFE indicates the stability of secondary structure. This is the basic requirement for a real pre-miRNA. The pMFE was calculated by RNAfold directly.

(2) pVStrc: The difference of the secondary structure of the two paired sequences resulting from mutations. The pVStrc is calculated by the following formula

$$\left(\sum\left|STRCa_{(pos)} \neq STRCb_{(pos)}\right|\right) \div \left(\sum\left|SEQa_{(pos)} \neq SEQb_{(pos)}\right|\right), \forall pos, pos \in (1,n)$$

(3) pVMFE: The difference of the MFE of the secondary structure of the two sequences aroused by the mutations. The pVMFE is calculated by the formula

$$\left(\left|pMFE_{(STRCa)} - pMFE_{(STRCb)}\right|\right) \div \left(\sum\left|SEQa_{(pos)} \neq SEQb_{(pos)}\right|\right), \forall pos, pos \in (1,n)$$

(4) pMutFeq: The mutation frequency of the sequence segment pair. Pre-miRNAs are usually highly conserved between different organisms. The pMutFeq is calculated by the formula

$$\left(\sum\left|SEQa_{(pos)} \neq SEQb_{(pos)}\right|\right) \div n, \forall pos, pos \in (1,n)$$

(5) pMatch，pMismatch，pDI，pBulge. As a good pre-miRNA candidate, there should also be a 22 mer region located in the stem to have the potential to be processed into a mature miRNA. The pMatch indicates the base pairing of the 22 mer putative mature miRNA. The pMismatch represents the frequency of non-pairing base pairs of the 22 mer putative mature miRNA (indicated by the size of the interior loops). The pDI represents the deletion and insertion frequencies of the 22 mer putative mature miRNA. The pBulge indicates the symmetry of the bulged loops. They are calculated by the following formula:

$$\max\left(\sum_{f=pos}^{pos+21}\left|STEM_{(f)} = "="\right|\right), \forall pos, pos \in (1,i) \text{ ; } \max\left(\sum_{f=pos}^{pos+21}\left|STEM_{(f)} = ":"\right|\right), \forall pos, pos \in (1,i) \text{ ;}$$

$$\max\left(\sum_{f=pos}^{pos+21}\left|(STEM_{(f)} = "." \text{ or } STEM_{(f)} = "-") \text{ and } STEM_{(f)} \neq "\wedge"\right|\right), \forall pos, pos \in (1,i) \text{ ;}$$

$$\max\left(\sum_{f=pos}^{pos+21}\left|STEMl_{(f)} = "\wedge"\right|\right), \forall pos, pos \in (1,i)$$

(6) The frequency of the 10 possible secondary structure elements (combinations of 2 adjacent symbols) in the pseudo code of the stem region (represented by the new syntax). The frequency is calculated by the formula $\left(\sum\left|STEM_{(pos,pos+1)} = "element"\right|\right) \div i, \forall pos, pos \in (1, i-1)$.

Quick scan the sequence segments for a pre-miRNA like hairpins

An algorithm was developed to quickly scan the genome pair-wise sequence to get the regions that have high potential to form a hairpin secondary structure. The algorithm moves a window (a pointer with 2 arms) along the sequence by 3-nucleotide steps. The sequence of the two arms was aligned by the algorithm. A score was assigned to the window-masked sequence based on the base pairing between the two arms. The details of the algorithm are described as below:

An algorithm based on the Smith-Waterman algorithm [1] was developed to quickly scan the genome pair-wise sequence to get the regions that have high potential to form a hairpin secondary structure. The algorithm includes two matrixes represented as H_1, H_2. To scan the whole genome, the algorithm moves a window (a pointer with 2 arms) along the sequence by 3-nucleotide steps, and assigns a score of the window-masked sequence. The score indicated the stability of the hypothesized fold-back. The length of the window is given by L (we set L to 71, which is the average length of the pre-miRNA). The two dimensions of the matrixes correspond to the two arms of the window, respectively. Two arms of the matrix were represented as $Al = Al_1, Al_2, Al_3, ..., Al_i$ and $Ar = Ar_1, Ar_2, Ar_3, ..., Ar_j$. An iterative process, the Smith-Waterman algorithm was introduced to find the most stable binding segments between the two arms. A new matrix measurement of the binding ability between the two arms was proposed based on the thermodynamic parameters of RNA [2, 3]. In this algorithm the pre-miRNA sequence was forced to fold into a hairpin secondary structure, which may overcome the problem of the most current RNA folding package to return a secondary structure containing multiple loops. The algorithm proceeds in three steps (supplement document Figure 2):

Step 1: Calculate the values of H_1, which represent the pairing state (paired or unpaired) of each base pair in the secondary structure of pre-miRNA. MiRFinder assigns a value of -3, -2 and -1 to GC, AU and GU base pairs, respectively, and 0 for other base pairs [2, 3]. Considering that no pre-miRNAs contain a hairpin loop smaller than a 3 mer, we first set $H(1,1) = H(1,2) = H(2,1) = 0$. Other values of H_1 are obtained by the following formula:

$$H_1(i,j) = \begin{cases} -3 & Al_i = "G", Ar_j = "C" \text{ or } Al_i = "C", Ar_j = "G" \\ -2 & Al_i = "A", Ar_j = "U" \text{ or } Al_i = "U", Ar_j = "A" \\ -1 & Al_i = "G", Ar_j = "U" \text{ or } Al_i = "U", Ar_j = "G" \\ 0 & else \end{cases} \quad \forall i, \forall j, i \in [0, L\text{-}1], j \in [0, L\text{-}1]$$

Step 2: Values of H_2 interpreted as $H_2(i,j)$ represent the maximum score of the sequence ending at Al_i to Ar_j respectively. Each value of $H_2(i,j)$ was determined by a trace back procedure for the consideration of each value of $H_2(m,n)$. The formula for calculation is described as follows:

$$H_2(i,j) = \begin{cases} \max\{\max\{H_1(i,j) + H_2(m,n) - E_{hairpin}(i+j-m-n-2)\}, 0\} & m+n=0 \\ \max\{\max\{H_1(i,j) + H_2(m,n) - E_{bulge}(i+j-m-n-2)\}, 0\} & i\text{-}m\text{-}1=0, j\text{-}n\text{-}1>0 \text{ or } i\text{-}m\text{-}1>0, j\text{-}n\text{-}1=0 \\ \max\{\max\{H_1(i,j) + H_2(m,n) - E_{interior}(i+j-m-n-2)\}, 0\} & i\text{-}m\text{-}1>0 \text{ and } j\text{-}n\text{-}1>0 \end{cases}$$

$$\forall i, \forall j, i \in [0, L\text{-}1], j \in [0, L\text{-}1], \forall m, \forall n, m \in [0, i-2], n \in [0, j-2], H_1(m,n) \neq 0$$

In which $E_{hairpin}(l)$, $E_{bulge}(l)$ and $E_{interior}(l)$ represent the punish score of the hairpin loop, bulge loop and interior loop, respectively, with a length of l. Every possible value of $E_{hairpin}(l)$, $E_{bulge}(l)$ and $E_{interior}(l)$ was calculated and stored in the punish score matrix [2, 3].

Punish score matrix

Destabilizing Energies by Size of Loop (M. Zuker, D. H. Mathews & D. H. Turner. Algorithms and Thermodynamics for RNA Secondary Structure Prediction: A Practical Guide In RNA Biochemistry and Biotechnology, 11-43)

SIZE	INTERNAL	BULGE	HAIRPIN
1	.	3.80	.
2	.	2.80	.
3	.	3.20	5.70
4	1.70	3.60	5.60
5	1.80	4.00	5.60
6	2.00	4.40	5.40
7	2.20	4.60	5.90
8	2.30	4.70	5.60
9	2.40	4.80	6.40
10	2.50	4.90	6.50
11	2.60	5.00	6.60
12	2.70	5.10	6.70
13	2.80	5.20	6.80
14	2.90	5.30	6.90
15	3.00	5.40	6.90
16	3.00	5.40	7.00
17	3.10	5.50	7.10
18	3.10	5.50	7.10
19	3.20	5.60	7.20
20	3.30	5.70	7.20
21	3.30	5.70	7.30
22	3.40	5.80	7.30
23	3.40	5.80	7.40
24	3.40	5.80	7.40
25	3.50	5.90	7.50
26	3.50	5.90	7.50
27	3.60	6.00	7.50
28	3.60	6.00	7.60
29	3.60	6.00	7.60
30	3.70	6.10	7.70

Step 3: Return the maximum score by $Score = \max\{H_2(i,j), 0\}, \forall i, \forall j, i \in [0, L-1], j \in [0, L-1]$.

Additional analysis: We recommend to do the following additional analysis

(1) Eliminate protein coding sequences by aligning these candidates to chicken mRNA sequences; (2) Use other RNA secondary prediction software to validate the genuine nature of the hairpin secondary structure; (3) eliminate all of the sequences associated with transposable elements, snRNAs, snoRNAs, tRNAs and rRNAs.

Supplementary Figure 1

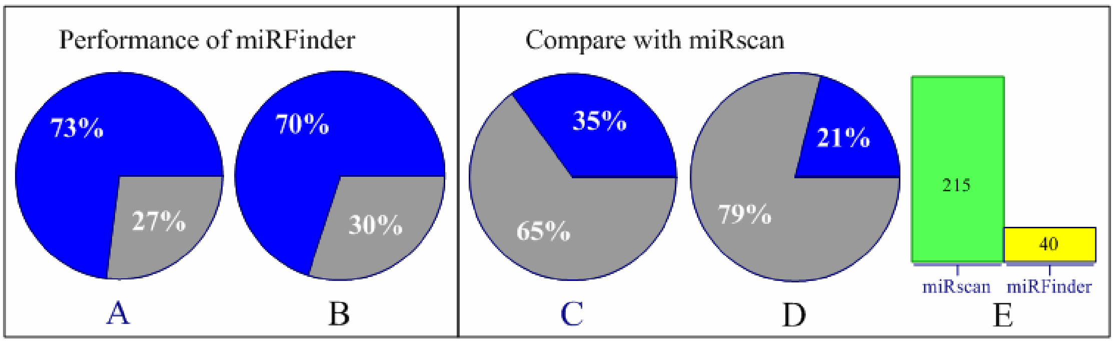

Figure 1: Performance comparison results between miRFinder and miRscan. It is shown that the miRFinder covers more experimentally confirmed chicken pre-miRNAs (A versus C) and produced more new candidates (B versus D) when compared to miRscan. The miRFinder also requires less running time (E; 40 seconds for miRFinder and 215 seconds for miRscan to process 530 sequence).

Supplementary Figure 2

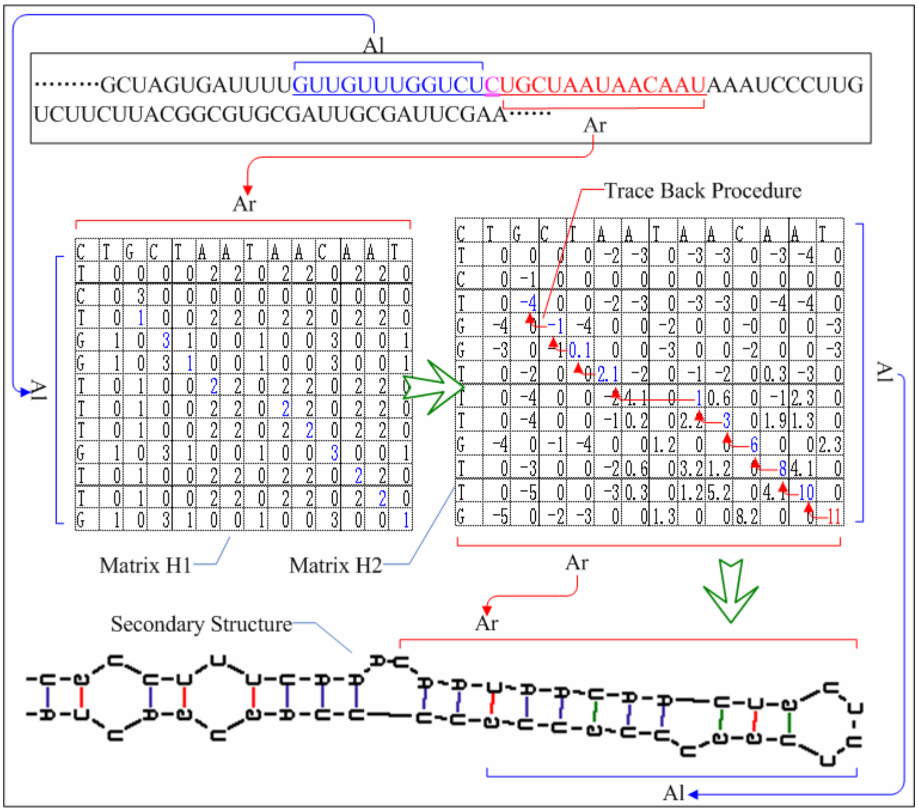

Figure 2: Quick scan of the sequence segments that can fold into a pre-miRNA like hairpin. The two dimensions of the matrix correspond to the two arms of the window. An iterative process like the Smith-Waterman algorithm was introduced into the algorithm to find the most stable binding segments between the two arms and return a punish score.

Supplementary Figure 3

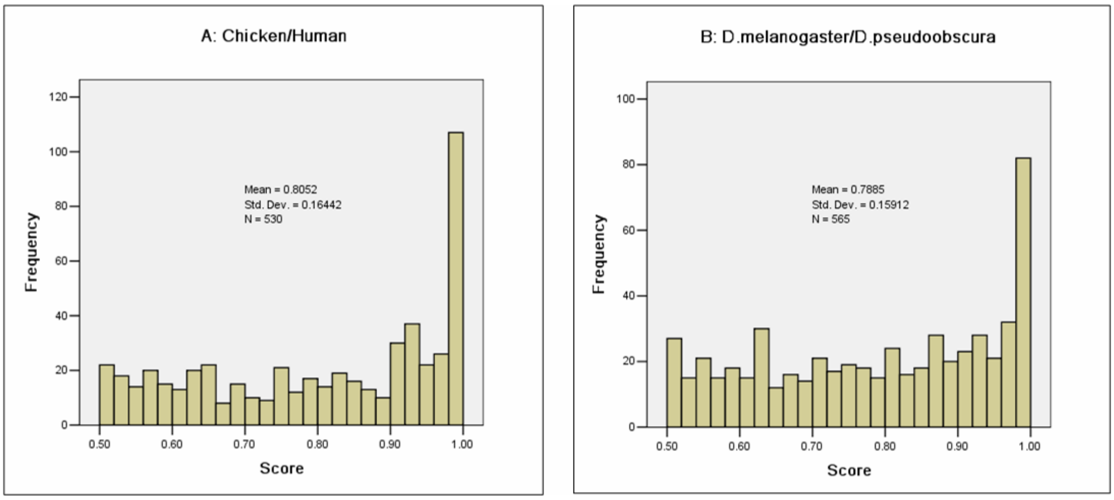

Figure 3: The distribution of the scores. (A) The score distribution of the hairpin from chicken/human pair-wise alignments. (B) The score distribution of the hairpin from D. melanogaster/D. pseudoobscura pair-wise alignments.

Supplementary Table 1

Table 1: The miRNAs and their potential functions

Name	Function
let-7	Regulates developmental timing in Caenorhabditis elegans [4]; degradation of lin-41 mRNA [5].
miR-1	Regulates connexin43 expression during skeletal muscle development [6]; regulates skeletal muscle proliferation and differentiation [7].
lin-4	Degradation lin-14 and lin-28 mRNA [5].
miR164a, miR164b	Controls petal number by regulating the transcript accumulation of CUC1 and CUC2 [8].
miR-15a, miR-16-1	Influence tumor growth [9]; down-regulates expression of Bcl2 expression in CLL (chronic lymphocytic leukemia) [10].
miR-122	Liver-specific, down-regulates CAT-1 [11]; regulation of lipid metabolism [12].
miR-214	Modulates hedgehog signaling to specify muscle cell fate [13].
miR-399	Down-regulates UBC mRNA accumulation [14].
miR-196	Upstream of Hoxb8 and Shh in limb development [15]; inhibits HOXB8 expression in myeloid differentiation of HL60 cells [16].
Mir-17-5p	Regulates breast cancer cell proliferation by inhibiting translation of AIB1 mRNA [17].
miR-206	Promotes muscle differentiation [18]; regulates connexin43 expression during skeletal muscle development [6].
miR-7b	Inhibits Fos translation [19].
miR-181	Regulates the homeobox protein Hox-A11 during mammalian myoblast differentiation [20]; regulates Tcl1 expression in chronic lymphocytic leukemia [21].
miR-29	Regulates Tcl1 expression in chronic lymphocytic leukemia [21].
miR156	Regulates expression of SPL3 [22].
mir-155	Related to the macrophage inflammatory response [23]. Regulates human angiotensin II type 1 receptor expression in fibroblasts [24]; oncogenic microRNA [25].
mir-143	Regulates adipocyte differentiation [26].
mir-133	Regulates skeletal muscle proliferation and differentiation [7].
mir-23b	Regulates expression of Hes1 gene in P19 cells [27].
mir-140	Targets histone deacetylase 4 in mouse cells [28].

Supplementary Table 2

Table 2: Software packages for miRNA identification applied in different species

Name	Description
miRScan	Using pair-wise genomic sequence and scoring the sequence segments based on the similarity of the conserved stem-loop to the 50 pairs of experimentally verified C. elegans/C. briggsae microRNA hairpins. [29]
MIRcheck	Takes as input the putative sequence, the putative structure, and the putative mature miRNA sequence. The parameters of the tool were adjusted to the properties such as base pairing, the length of the hairpin and the asymmetry of the loops of plant miRNAs. [30]
miRseeker	Applied in Drosophila melanogaster and D. pseudoobscura genomes. The methodology of this tool is the comparison of a 20mer within a predicted secondary structure against a set of parameters that describe the majority of previously known plant miRNAs and their hairpin structure such as the characteristic of extended stem-loop structure and pattern of nucleotide divergence of miRNAs. [31]
findMiRNA	Applied in Arabidopsis thaliana. Relies on the complementarity between existing miRNAs and their targets and the complementarity of the adjacent sequence that would enable stem-loop formation in an RNA molecule consistent with the known structures of miRNA precursors. [32]
PalGrade	Can identify conserved and nonconserved miRNAs in human genome. The pipeline of the method is: scan the conserved stem-loops in the genome; remove the conserved, repetitive and protein-coding regions. [33]
MiRAlign	The basic methodology employed in this tool is sequence and structure comparison. The tool can be used to find miRNAs similar (homolog) to the experimentally confirmed miRNAs. [34]
ProMiR	This tool introduced a probabilistic co-learning model, the hidden Markov model (HMM), to classify miRNA genes based on their pair-wise aligned sequences. Taking advantage from the new methodology (HMM), the tool yields false-positive rate as low as 4.00%. However, the sensitivity of the tool is relative poor (only 73.00%). [35]
Triplet-SVM	The tool improved its performance by introduction of a new syntax. The syntax encoded the local contiguous structure/sequence features using 32 triplet elements, a nucleotide type and three continuous substructures. The frequency of these elements provide a set of promising parameters. [36]
RNAmicro	A SVM based program by introducing several descriptors. For example the length of the stem, the length of the hairpin, the G+C content and etc. The tool can be used to recognizing microRNA precursors in multiple sequence alignments and have been successfully applied to recent genomewide RNAz surveys of mammals, urochordates, and nematodes. [37]
miRNA SVM	SVM based miRNAs discovery tool. The tool improved performance by introduce a prediction algorithm of 5' Drosha processing sites in hairpins. The classifier can correctly predicts the processing site for 50% of known human 5' miRNAs. miRNA SVM used about 18 features such as precursor length and loop size, distance from processing site to the loop start and etc. [38]
miPred	The tool characterizes a pre-miRNA at the dinucleotide sequence, hairpin folding, non-linear statistical thermodynamics and topological levels. The SVM classifier model the tool used was trained on 200 human pre-miRs and 400 pseudo hairpins, and achieved 93.50% accuracy. [39]

References

1. Smith TF, Waterman MS: **Identification of common molecular subsequences**. *J Mol Biol* 1981, **147**(1):195-197.
2. Sugimoto N, Nakano S, Katoh M, Matsumura A, Nakamuta H, Ohmichi T, Yoneyama M, Sasaki M: **Thermodynamic parameters to predict stability of RNA/DNA hybrid duplexes**. *Biochemistry* 1995, **34**(35):11211-11216.
3. Mathews DH, Sabina J, Zuker M, Turner DH: **Expanded sequence dependence of thermodynamic parameters improves prediction of RNA secondary structure**. *J Mol Biol* 1999, **288**(5):911-940.
4. Abbott AL, Alvarez-Saavedra E, Miska EA, Lau NC, Bartel DP, Horvitz HR, Ambros V: **The let-7 MicroRNA family members mir-48, mir-84, and mir-241 function together to regulate developmental timing in Caenorhabditis elegans**. *Developmental cell* 2005, **9**(3):403-414.
5. Bagga S, Bracht J, Hunter S, Massirer K, Holtz J, Eachus R, Pasquinelli AE: **Regulation by let-7 and lin-4 miRNAs results in target mRNA degradation**. *Cell* 2005, **122**(4):553-563.
6. Anderson C, Catoe H, Werner R: **MIR-206 regulates connexin43 expression during skeletal muscle development**. *Nucleic acids research* 2006, **34**(20):5863-5871.
7. Chen JF, Mandel EM, Thomson JM, Wu Q, Callis TE, Hammond SM, Conlon FL, Wang DZ: **The role of microRNA-1 and microRNA-133 in skeletal muscle proliferation and differentiation**. *Nat Genet* 2006, **38**(2):228-233.
8. Baker CC, Sieber P, Wellmer F, Meyerowitz EM: **The early extra petals1 mutant uncovers a role for microRNA miR164c in regulating petal number in Arabidopsis**. *Curr Biol* 2005, **15**(4):303-315.
9. Bottoni A, Piccin D, Tagliati F, Luchin A, Zatelli MC, degli Uberti EC: **miR-15a and miR-16-1 down-regulation in pituitary adenomas**. *Journal of cellular physiology* 2005, **204**(1):280-285.
10. Cimmino A, Calin GA, Fabbri M, Iorio MV, Ferracin M, Shimizu M, Wojcik SE, Aqeilan RI, Zupo S, Dono M *et al*: **miR-15 and miR-16 induce apoptosis by targeting BCL2**. *Proceedings of the National Academy of Sciences of the United States of America* 2005, **102**(39):13944-13949.
11. Chang J, Nicolas E, Marks D, Sander C, Lerro A, Buendia MA, Xu C, Mason WS, Moloshok T, Bort R *et al*: **miR-122, a mammalian liver-specific microRNA, is processed from hcr mRNA and may downregulate the high affinity cationic amino acid transporter CAT-1**. *RNA biology* 2004, **1**(2):106-113.
12. Esau C, Davis S, Murray SF, Yu XX, Pandey SK, Pear M, Watts L, Booten SL, Graham M, McKay R *et al*: **miR-122 regulation of lipid metabolism revealed by in vivo antisense targeting**. *Cell metabolism* 2006, **3**(2):87-98.
13. Flynt AS, Li N, Thatcher EJ, Solnica-Krezel L, Patton JG: **Zebrafish miR-214 modulates Hedgehog signaling to specify muscle cell fate**. *Nat Genet* 2007.
14. Fujii H, Chiou TJ, Lin SI, Aung K, Zhu JK: **A miRNA involved in phosphate-starvation response in Arabidopsis**. *Curr Biol* 2005, **15**(22):2038-2043.
15. Hornstein E, Mansfield JH, Yekta S, Hu JK, Harfe BD, McManus MT, Baskerville S, Bartel DP, Tabin CJ: **The microRNA miR-196 acts upstream of Hoxb8 and Shh in limb development**.

Nature 2005, **438**(7068):671-674.

16. Kawasaki H, Taira K: **MicroRNA-196 inhibits HOXB8 expression in myeloid differentiation of HL60 cells**. *Nucleic acids symposium series (2004)* 2004(48):211-212.
17. Hossain A, Kuo MT, Saunders GF: **Mir-17-5p regulates breast cancer cell proliferation by inhibiting translation of AIB1 mRNA**. *Molecular and cellular biology* 2006, **26**(21):8191-8201.
18. Kim HK, Lee YS, Sivaprasad U, Malhotra A, Dutta A: **Muscle-specific microRNA miR-206 promotes muscle differentiation**. *The Journal of cell biology* 2006, **174**(5):677-687.
19. Lee HJ, Palkovits M, Young WS, 3rd: **miR-7b, a microRNA up-regulated in the hypothalamus after chronic hyperosmolar stimulation, inhibits Fos translation**. *Proceedings of the National Academy of Sciences of the United States of America* 2006, **103**(42):15669-15674.
20. Naguibneva I, Ameyar-Zazoua M, Polesskaya A, Ait-Si-Ali S, Groisman R, Souidi M, Cuvellier S, Harel-Bellan A: **The microRNA miR-181 targets the homeobox protein Hox-A11 during mammalian myoblast differentiation**. *Nature cell biology* 2006, **8**(3):278-284.
21. Pekarsky Y, Santanam U, Cimmino A, Palamarchuk A, Efanov A, Maximov V, Volinia S, Alder H, Liu CG, Rassenti L *et al*: **Tcl1 expression in chronic lymphocytic leukemia is regulated by miR-29 and miR-181**. *Cancer research* 2006, **66**(24):11590-11593.
22. Wu G, Poethig RS: **Temporal regulation of shoot development in Arabidopsis thaliana by miR156 and its target SPL3**. *Development (Cambridge, England)* 2006, **133**(18):3539-3547.
23. O'Connell R M, Taganov KD, Boldin MP, Cheng G, Baltimore D: **MicroRNA-155 is induced during the macrophage inflammatory response**. *Proceedings of the National Academy of Sciences of the United States of America* 2007.
24. Martin MM, Lee EJ, Buckenberger JA, Schmittgen TD, Elton TS: **MicroRNA-155 regulates human angiotensin II type 1 receptor expression in fibroblasts**. *J Biol Chem* 2006, **281**(27):18277-18284.
25. Tam W, Dahlberg JE: **miR-155/BIC as an oncogenic microRNA**. *Genes, chromosomes & cancer* 2006, **45**(2):211-212.
26. Esau C, Kang X, Peralta E, Hanson E, Marcusson EG, Ravichandran LV, Sun Y, Koo S, Perera RJ, Jain R *et al*: **MicroRNA-143 regulates adipocyte differentiation**. *J Biol Chem* 2004, **279**(50):52361-52365.
27. Kimura H, Kawasaki H, Taira K: **Mouse microRNA-23b regulates expression of Hes1 gene in P19 cells**. *Nucleic acids symposium series (2004)* 2004(48):213-214.
28. Tuddenham L, Wheeler G, Ntounia-Fousara S, Waters J, Hajihosseini MK, Clark I, Dalmay T: **The cartilage specific microRNA-140 targets histone deacetylase 4 in mouse cells**. *FEBS letters* 2006, **580**(17):4214-4217.
29. Lim LP, Lau NC, Weinstein EG, Abdelhakim A, Yekta S, Rhoades MW, Burge CB, Bartel DP: **The microRNAs of Caenorhabditis elegans**. *Genes & development* 2003, **17**(8):991-1008.
30. Jones-Rhoades MW, Bartel DP: **Computational identification of plant microRNAs and their targets, including a stress-induced miRNA**. *Molecular cell* 2004, **14**(6):787-799.
31. Lai EC, Tomancak P, Williams RW, Rubin GM: **Computational identification of Drosophila microRNA genes**. *Genome biology* 2003, **4**(7):R42.
32. Adai A, Johnson C, Mlotshwa S, Archer-Evans S, Manocha V, Vance V, Sundaresan V:

Computational prediction of miRNAs in Arabidopsis thaliana. *Genome research* 2005, **15**(1):78-91.

33. Bentwich I, Avniel A, Karov Y, Aharonov R, Gilad S, Barad O, Barzilai A, Einat P, Einav U, Meiri E *et al*: **Identification of hundreds of conserved and nonconserved human microRNAs**. *Nature genetics* 2005, **37**(7):766-770.
34. Wang X, Zhang J, Li F, Gu J, He T, Zhang X, Li Y: **MicroRNA identification based on sequence and structure alignment**. *Bioinformatics (Oxford, England)* 2005, **21**(18):3610-3614.
35. Nam JW, Shin KR, Han J, Lee Y, Kim VN, Zhang BT: **Human microRNA prediction through a probabilistic co-learning model of sequence and structure**. *Nucleic acids research* 2005, **33**(11):3570-3581.
36. Xue C, Li F, He T, Liu GP, Li Y, Zhang X: **Classification of real and pseudo microRNA precursors using local structure-sequence features and support vector machine**. *BMC Bioinformatics* 2005, **6**:310.
37. Hertel J, Stadler PF: **Hairpins in a Haystack: recognizing microRNA precursors in comparative genomics data**. *Bioinformatics (Oxford, England)* 2006, **22**(14):e197-202.
38. Helvik SA, Snove O, Jr., Saetrom P: **Reliable prediction of Drosha processing sites improves microRNA gene prediction**. *Bioinformatics (Oxford, England)* 2007, **23**(2):142-149.
39. Kwang Loong SN, Mishra SK: **De Novo SVM Classification of Precursor MicroRNAs from Genomic Pseudo Hairpins Using Global and Intrinsic Folding Measures**. *Bioinformatics (Oxford, England)* 2007.
